# Effect of intermittent exposure to ethanol and MDMA during adolescence on learning and memory in adult mice

**DOI:** 10.1186/1744-9081-8-32

**Published:** 2012-06-20

**Authors:** Antonio Vidal-Infer, Maria A Aguilar, Jose Miñarro, Marta Rodríguez-Arias

**Affiliations:** 1 Unidad de Investigación Psicobiología de las Drogodependencias, Departamento de Psicobiología, Facultad de Psicología, Universitat de Valencia, Avda. Blasco Ibáñez 21, 46010, Valencia, Spain

**Keywords:** Ethanol, MDMA, Hebb Williams maze, Learning, Memory

## Abstract

****Background**:**

Heavy binge drinking is increasingly frequent among adolescents, and consumption of 3,4-methylenedioxymethamphetamine (MDMA) is often combined with ethanol (EtOH). The long-lasting effects of intermittent exposure to EtOH and MDMA during adolescence on learning and memory were evaluated in adult mice using the Hebb-Williams maze.

****Methods**:**

Adolescent OF1 mice were exposed to EtOH (1.25 g/kg) on two consecutive days at 48-h intervals over a 14-day period (from PD 29 to 42). MDMA (10 or 20 mg/kg) was injected twice daily at 4-h intervals over two consecutive days, and this schedule was repeated six days later (PD 33, 34, 41 and 42), resulting in a total of eight injections. Animals were initiated in the Hebb-Williams maze on PND 64. The concentration of brain monoamines in the striatum and hippocampus was then measured.

****Results**:**

At the doses employed, both EtOH and MDMA, administered alone or together, impaired learning in the Hebb-Williams maze, as treated animals required more time to reach the goal than their saline-treated counterparts. The groups treated during adolescence with EtOH, alone or plus MDMA, also presented longer latency scores and needed more trials to reach the acquisition criterion score. MDMA induced a decrease in striatal DA concentration, an effect that was augmented by the co-administration of EtOH. All the treatment groups displayed an imbalance in the interaction DA/serotonin.

****Conclusions**:**

The present findings indicate that the developing brain is highly vulnerable to the damaging effects of EtOH and/or MDMA, since mice receiving these drugs in a binge pattern during adolescence exhibit impaired learning and memory in adulthood.

## **Background**

MDMA (3,4-methylenedioxymethamphetamine) users also consume ethanol frequently (EtOH) [1,2]. For example, [3] reported that 85% of those attending rave parties consumed both EtOH and MDMA. Similarly, heavy binge drinking is becoming increasingly common among teenagers in the USA and Europe [4-6]. In a survey of Spanish adolescents, 49.6% of those who had consumed alcohol in the previous month reported getting drunk during binges. Among those who consumed ecstasy, 98% admitted taking it with alcohol. Similarly, use of ecstasy is more common among adolescents who drink alcohol (2.5%) [7]. Research with human adolescents has provided clear evidence that alcohol abuse during the teenage years has deleterious effects, with alcohol-related problems and neurological deficits being more prevalent among adolescents that abuse alcohol [5,8].

EtOH is an allosteric modulator of many transmembrane receptors [9]. Functionally, it acts primarily as a CNS depressant, potentiating the action of GABA at the GABAA receptor [10]. MDMA, on the other hand, causes a rapid efflux of dopamine (DA) and serotonin (5-HT) in several brain areas immediately after it is administered, including the striatum and nucleus accumbens (NAc), [11]. Research has only recently begun to focus on EtOH–MDMA interactions in animal models [12-15], and the studies undertaken have been characterized by a great inconsistency in the treatment schedules employed and the time at which measurements were taken. EtOH has been shown to increase blood concentrations of MDMA, and more intensely in the striatum and cortex than in the hippocampus [16]. On the other hand, levels of alcohol dehydrogenase 2, which metabolizes EtOH to acetaldehyde, have been found to be 35% lower in MDMA-treated rats than in controls [17].

EtOH modifies many of the effects of MDMA, and studies suggest that the interaction between the two drugs depends on the dose, administration regimen and ambient temperature in question [18,19]. Few studies to date have evaluated chronic exposure to both EtOH and MDMA. In recent experiments, we have observed that MDMA administration during adolescence induces a specific behavioral and neurochemical profile in adult animals when combined with a pattern of EtOH administration that models binge drinking. This is evidence that the interaction of these two drugs in the adolescent brain produces lasting effects [20]. In addition, exposure to MDMA during adolescence increased the anxyogenic response and decreased concentrations of DA in the striatum. EtOH increased these effects while undermining the hyperthermic response induced by MDMA. In the study in question, passive avoidance was affected only when EtOH was administered alone.

Clinical and experimental studies have provided evidence of the special sensitivity of the adolescent brain to some effects of EtOH, such as memory impairment [21] and EtOH-induced brain damage [22]. Adolescents are less sensitive to EtOH-induced motor impairments [23] and loss of righting reflex [24] but are more sensitive to EtOH-induced hypothermia [25] and hippocampal-dependent memory impairments [26], although the opposite effect has also been reported [27,28]. A possible explanation for these discrepant results is that adolescents learn the spatial Morris water maze task more slowly than adults, although EtOH is thought to impair spatial memory in both age groups [29]. We have previously observed that intermittent administration of EtOH during adolescence enhances neural cell death in several brain regions (neocortex, hippocampus and cerebellum) and produces long-lasting neurobehavioral impairments in conditional discrimination learning, motor learning and discrimination between novel and familiar objects [30].

Based on the abovementioned studies, we hypothesized that intermittent EtOH and/or MDMA intoxication during adolescence would have long-lasting consequences for memory and learning. The aim of the present study was to employ the Hebb-Williams maze to investigate how MDMA mediates the long-term consequences of exposure to EtOH during adolescence for memory function. In order to clarify whether or not the effects of exposure to drugs during adolescence are related with the neurotoxic damage that they produce, we determined the concentration of dopamine and serotonin and their metabolites in the striatum and hippocampus of animals exposed to MDMA alone or plus EtOH.

## **Methods**

### **Subjects**

A total of 78 male mice of the OF1 strain (CHARLES RIVER, Barcelona, Spain) were employed in the study. Animals were 21 days old on arrival at the laboratory and were all housed under standard conditions in groups of four (cage size 28 x 28 x 14.5 cm), at a constant temperature (21+ 2°C), with a reversed light schedule (white lights on 19:30–07:30 h) and food and water available ad libitum (except during the behavioral test). All procedures involving the mice and their care complied with national, regional and local laws and regulations, and with European Community Council Directives (86/609/EEC, 24 November 1986).

### **Drug treatment and experimental design**

The doses of EtOH [20,31-33] and MDMA [34-39] were based on those used in previous studies. Animals were injected i.p with volumes of 0.01 ml/g MDMA (±3,4-methylenedioxymetamphetamine hydrochloride, Laboratorios Lipomed AG, Switzerland) and EtOH in a volume of 0.02 ml/g. The control group was injected with physiological saline (NaCl 0.9%), which was also used for dissolving the drugs. The groups receiving both EtOH and MDMA were administered each substance in a separate injection. The EtOH dose employed (1.25 g/kg) induced a blood concentration of 0.9 mg/ml in OF1 adolescent mice 5 min after administration. In an adolescent human, this dose would correspond with 33 g of EtOH, which represents two or three alcoholic drinks (taking into account that one alcoholic drink contains 13.7 g of EtOH).

After an acclimatization period of 8 days, animals were divided into 6 groups: two groups received physiological saline (Sal, n = 8) or 1.25 g/kg of EtOH (E1.25, n = 8) in a schedule in which injections were administered twice daily (with a 4-hour interval) on two consecutive days followed by an interval of two “drug-free” days, over a two-week period (a total of 16 doses). Another two groups received 10 or 20 mg/kg of MDMA (M10, n = 8; M20, n = 8) in a pattern in which injections were given twice daily (with a 4 hour interval) on two consecutive days, with an interval of six days without injections, over a two-week period (a total of 8 doses). The last two groups received 1.25 g/kg of EtOH and 10 or 20 mg/kg of MDMA (E1.25 + M10, n = 8; E1.25 + M20, n = 8) in a schedule in which adolescent animals were injected twice daily with EtOH on PND 29, 30, 37 and 38 and with EtOH plus MDMA on PND 33, 34, 41 and 42. Behavioral tests were performed three weeks after treatment had finalized (PND 64 to 75). In this way, each mouse received eight drug administrations that simulated a binge pattern characteristic of that seen in human adolescents and young adults [40]. A more detailed description of the experimental procedure is presented in Table 1.

**Table 1:** Experimental procedure

**Groups / PND**	**29**	**30**	**31-32**	**33**	**34**	**35-36**	**37**	**38**	**39-40**	**41**	**42**	**43-63**	**64**
**Sal**	FS/FS	FS/FS		FS/FS	FS/FS		FS/FS	FS/FS		FS/FS	FS/FS	3 weeks without treatment	Hebb--‐ Williams maze
**M10**				M10/M10	M10/M10					M10/M10	M10/M10		
**M20**				M20/M20	M20/M20					M10/M10	M10/M10		
**E1.25**				E1.25/E1.25	E1.25/E1.25	E1.25/E1.25	E1.25/E1.25			E1.25/E1.25	E1.25/E1.25		
**E1.25+M10**				E1.25+M10/E1.25+M10	E1.25+M10/E1.25+M10	E1.25/E1.25	E1.25/E1.25			E1.25+M10/E1.25+M10	E1.25+M10/E1.25+M10		
**E1.25+M20**				E1.25+M20/E1.25+M20	E1.25+M20/E1.25+M20	E1.25/E1.25	E1.25/E1.25			E1.25+M20/E1.25+M20	E1.25+M20/E1.25+M20		

### **Procedure and apparatus**

The maze we used in our experiments is made of black plastic and is 60 cm wide × 60 cm long × 10 cm high. It contains a start box and a goal box (both 14 cm wide × 9 cm long) which are positioned at diagonally opposite corners. The maze contains cold water at a wading depth (15°C, 3.5 cm high), while the goal box is stocked with fresh dry tissue. Several maze designs are produced by fixing different arrangements of barriers to a clear plastic ceiling. This apparatus allows the cognitive process of routed learning and the motivation of water escape to be measured.

The procedure we followed was based on that employed by Galsworthy et al. [41], in which mice must navigate the maze and cross from the wet start box to the dry goal box in order to escape the cold water. Animals underwent a 5-min habituation period (dry sand, no barriers) on day 1 and undertook problem A on day 2 and problem D on day 3 (4 trials/day) (practice mazes). Mice were subsequently submitted to mazes 1, 5, 3, 4 and 8 on separate days on which 8 trials took place (see [42] for all maze designs). The time limit for reaching the goal box was 5 min, after which the mouse was guided to the box. The following measurements were recorded: acquisition criterion score, considered to be completion of the task in less than 60 s in two consecutive sessions; total latency score (the sum of the latencies in all the problem trials in each maze); latency for reaching the goal in the 8th trial; and error scores, for which a similar total was used (where “error” was considered as entering the error zone specified by [42]).

Following the Stanford and Brown classification (2003), the mazes were defined as easy (1, 3 and 4) or difficult (5 and 8).

#### ***Analysis of biogenic amines***

A different set of animals (n = 5 in each group) was exposed to the same treatment schedule, but on the day corresponding with their introduction to the maze, were sacrificed by cervical fracture following a procedure similar to that described in Daza-Losada et al. [34]. Within 2 min, their brains were removed and placed on an ice-cold plate. The striatum and hippocampus were removed, frozen on dry ice, and stored at −80°C. The tissue was thawed, weighed and then homogenized in 200 μl of perchloric acid (0.1 N) using ultrasounds. The homogenate was centrifuged at 14,000 rpm for 30 min. The supernatant was divided into aliquots for the analysis of biogenic amines. Using a high performance liquid chromatograph (Agilent 1100 series HPLC) Dopamine (DA), dihydroxyphenyl acetic acid (DOPAC), serotonin (5-HT) and 5-hydroxyindole acetic acid (5-HIAA) were analyzed in the striatum and 5-HT and 5-HIAA were analyzed in the hippocampus. Samples were applied to a column (ZORBAX Eclipse XDB-C8 46 × 150 mm, 5 μm; Agilent Zorbax High Pressure Cartridge Guard-column). A mobile phase consisting of a 800 ml solution of sodium acetate (0.01 M), a 500 ml solution of citric acid (0.01 M), ethylenediaminetetraacetic acid disodium salt dehydrate (EDTA, 148 mg) and methanol (255 ml) was passed through the column at a constant flow of 1 ml/min. The HPLC was maintained at a constant temperature (21 ± 1°C). Analytes were oxidized on a glassy carbon electrode maintained at 300 mV (450 mV for HVA detection) against an Ag/AgCl reference electrode (BAS). The complete separation of biogenic amines was achieved in 25 min. Data were collected and analyzed using the Merk-Hitachi software package (Model D-7000).

### **Statistical analysis**

The data of the Hebb-Williams maze were analyzed using an ANOVA with two “between” subject variables - Ethanol, with two levels (0 and 1.25 mg/kg), and MDMA, with three levels (0, 10 and 20 mg/kg) - and one “within” subject variable- level of difficulty of the maze, with two levels (easy and difficult). Maximum latencies were scored by individuals unable to complete the task within the time limit. Latency values in the Hebb Williams maze were transformed to log scores in order to normalize the data.

An ANOVA of the latency to reach the goal and the number of errors made during the eight trials was performed with the same between variables and a within variable - “trial”, with eight levels - for both easy and difficult mazes.

Monoamine concentration was analyzed using a mixed ANOVA with the two mentioned “between” subject variables.

## **Results**

### **Hebb Williams maze**

**Figure 1:**
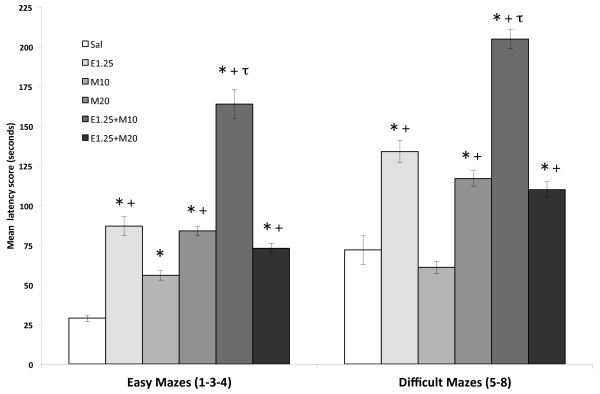
**Effects of intermittent ethanol and MDMA administration during adolescence on the mean latency score in the Hebb-Williams maze.** The mazes were classified as easy (1, 3 and 4) or difficult (5 and 8). Mice were treated with Saline (Sal), 10 or 20 mg/kg of MDMA (M10 and M20), 1.25 g/kg of ethanol (E1.25), or 1.25 g/kg of ethanol + 10 or 20 mg/kg of MDMA (E1.25 + M10 and E1.25 + M20). Data are presented as mean values ± S.E.M. Differences with respect to the saline group * p < 0.001; with respect to the M10 group + p < 0.001; with respect to the E1.25 and e.125 + M20 group τ p < 0.00.

The ANOVA for the mean of the total latency score (Figure 1) revealed an effect of the variable Level of difficulty [F (1,42) = 134.225; p < 0.001], as more time was employed in the difficult mazes than in the easy ones (p < 0.001). The interaction Maze × Ethanol × MDMA also had a significant effect [F (2,42) = 3.256; p < 0.05]. In the easy mazes, all the treatment groups employed more time to reach the goal than those receiving saline (p < 0.001 in all cases). In the difficult mazes, Saline and M10 groups had shorter latencies than the rest of the groups (p < 0.001). In both mazes, the M10 group showed shorter latencies than the E1.25 and M20 groups and both the groups receiving EtOH plus MDMA (p < 0.001 in all cases). Moreover, longer latencies were displayed by the groups treated with E1.25 + M10 than those treated only with EtOH or EtOH plus M20 (P < 0.001 in both mazes).

**Figure 2:**
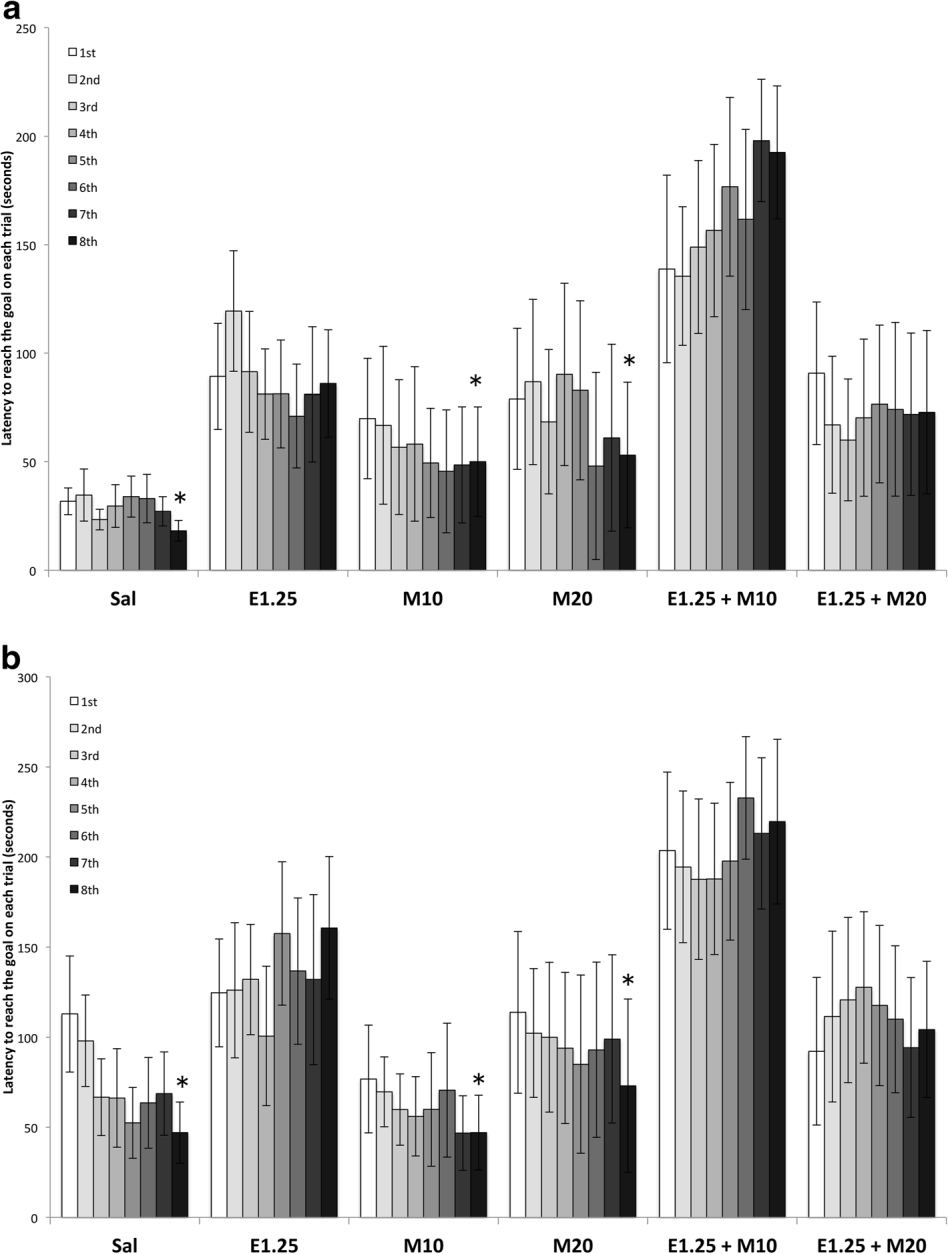
**Effects of intermittent ethanol and MDMA administration during adolescence on latency to reach the goal on each trail in the Hebb-Williams maze (2a for the easy and 2b for the difficult mazes).** The mazes were classified as easy (1, 3 and 4) or difficult (5 and 8). Mice were treated with Saline (Sal), 10 or 20 mg/kg of MDMA (M10 and M20), 1.25 g/kg of ethanol (E1.25), or 1.25 g/kg of ethanol + 10 or 20 mg/kg of MDMA (E1.25 + M10 and E1.25 + M20). Data are presented as mean values ± S.E.M. Differences with respect to the first trial * p < 0.05 in the easy and 0.001 in the difficult.

The ANOVA for the latency to reach the goal in each trial (Figure 2a and 2b) revealed a significant effect of the interaction Trial × Ethanol in the easy [F (7,294) = 2.636; p < 0.01] and difficult mazes [F (7,294) = 3.737; p < 0.001]. In the easy mazes, longer latencies were observed in the groups treated with EtOH in all the trials but the second (p < 0.03 for the first and third and p < 0.01 for the remaining trials). In the difficult mazes, the groups treated with EtOH also showed longer latencies in the fifth (p < 0.01), sixth (p < 0.01), seventh (p < 0.04) and eighth trials (p < 0.01). In addition, the groups that did not receive EtOH presented a significantly shorter latency in the last trial than in the first in both types of mazes (p < 0.05 for the easy and p < 0.001 for the difficult). However, changes were not detected in the groups treated with EtOH.

**Figure 3:**
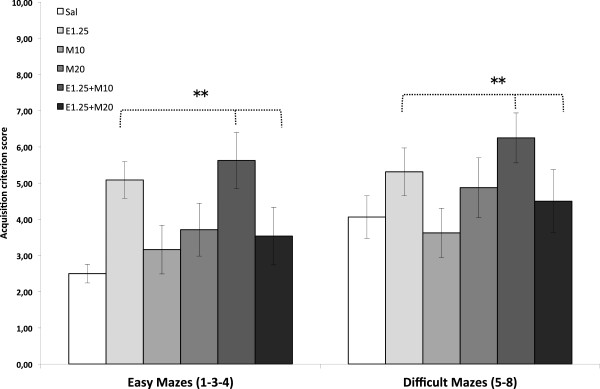
**Effects of intermittent ethanol and MDMA administration during adolescence on the acquisition criterion score in the Hebb-Williams maze.** The mazes were classified as easy (1, 3 and 4) or difficult (5 and 8). Mice were treated with Saline (Sal), 10 or 20 mg/kg of MDMA (M10 and M20), 1.25 g/kg of ethanol (E1.25), or 1.25 g/kg of ethanol + 10 or 20 mg/kg of MDMA (E1.25 + M10 and E1.25 + M20). The acquisition criterion score is considered to be the completion of the task in less than 60 s in two consecutive sessions. Data are presented as mean values ± S.E.M. Differences with respect to the non-ethanol-treated groups ** p < 0.01.

The ANOVA for the Acquisition criterion score (Figure 3), which represented the number of trials necessary for the task to be completed in less than 60 s in two consecutive sessions, revealed an effect of the variable Level of difficulty [F (1,42) = 13.645; p < 0.001], as the mice employed more time in the difficult mazes than the easy ones (p < 0.001). Administration of EtOH also showed a significant effect [F (1,42) = 6.373; p < 0.01], as the groups treated with EtOH needed more trials to complete the task (p < 0.01). This effect was the result of the high number of trials necessary for the E1.25 and E1.25 + M10 groups to meet this criterion in the easy mazes (p < 0.03).

**Figure 4:**
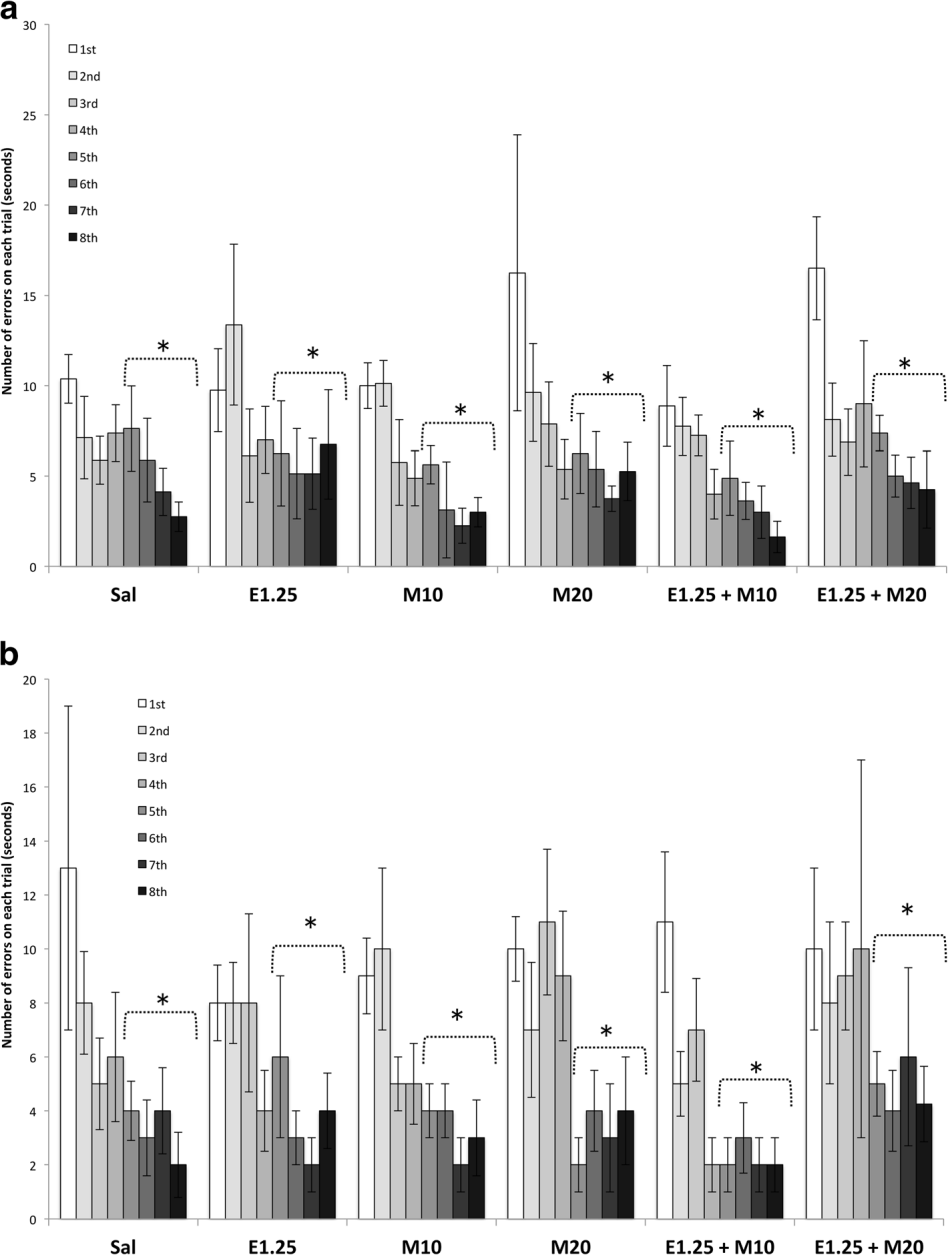
**Effects of intermittent ethanol and MDMA administration during adolescence on number of errors on each trail in the Hebb-Williams maze (4a for the easy and 4b for the difficult mazes).** The mazes were classified as easy (1, 3 and 4) or difficult (5 and 8). Mice were treated with Saline (Sal), 10 or 20 mg/kg of MDMA (M10 and M20), 1.25 g/kg of ethanol (E1.25), or 1.25 g/kg of ethanol + 10 or 20 mg/kg of MDMA (E1.25 + M10 and E1.25 + M20). Data are presented as mean values ± S.E.M. Differences with respect to first, second, third and fourth trials * p < 0.001.

The ANOVA for the total number of errors revealed an effect of the variable Level of difficulty [F (1,42) = 7.119; p < 0.001], as a higher number of errors were observed in the difficult mazes than in the easy ones (p < 0.001). The ANOVA for the number of errors in each trial (Figure 4a and 4b) showed that all the groups committed significantly fewer errors in trials 5, 6, 7 and 8 than in trials 1, 2, 3 and 5 in both easy and difficult mazes (p < 0.001 in all cases).

### **Brain monoamines**

The brain monoamine data are presented in Table 2. The ANOVA performed for the striatal levels of DA showed a significant effect of the interaction Alcohol × Dose of MDMA [F (2,30) = 3.955; p < 0.03]. Lower levels of DA were detected in the M20, E1.25 + M10 and E1.25 + M20 groups than in the saline and M10 groups (p < 0.001, in all cases).

**Table 2:** Effects of intermittent ethanol and MDMA administration during adolescence on the concentration of brain monoamines in the striatum and hippocampus in mice

	**Sal**	**M10**	**M20**	**E1.25**	**E1.25+M10**	**E1.25+M20**
***Striatum***						
**DA**	12364±1015	13315±981	7454±713***	11962±1176	9471±1929***	9850±1041***
**DOPAC**	1519±210	927±102	719±53++	1082±119	1069±202	765±106++
**DATurnover**	0.12±0.02	0.07±0.001**	0.1±0.01	0.09±0.01*	0.11±0.01	0.08±0.001
**5-HT**	924±76	1110±95	825±72	1247±98+	1128±167+	1200±289+
**5-HIIA**	547±86	441±16	450±47	590±101	859±234	507±57
**5-HT Turnover**	0.59±0.07	0.4±0.02	0.6±0.06	0.51±0.08	0.75±0.17	0.53±0.18
***Hippocampus***						
**5--‐HT**	534±37	570±67	489±179	959±207+	560±48+	635±56+
**5--‐HIIA**	504±85	613±111	541±65	1109±375	496±69	637±37

Animals were treated during adolescence with Saline (Sal), 10 mg/kg of MDMA (M10), 20 mg/kg of MDMA (M20), 1.25 mg/kg of ethanol (E1.25), 1.25 mg/kg of ethanol+10 mg/kg of MDMA (E1.25+M10), or 1.25 mg/kg of ethanol+20 mg/kg of MDMA (E1.25+M20). Data are presented in means with ±S.E.M. Differences with respect to the saline group *p< 0.05, ** p< 0.02, *** p< 0.001; differences with respect to non-EtOH treated groups + p< 0.03; differences with respect to non-MDMA treated groups ++ p< 0.001.

Striatal DOPAC levels showed a significant effect of the variable MDMA [F (2, 30) = 7.666; p < 0.003]. Lower levels of this metabolite were detected in the groups treated with 20 mg/kg of MDMA (M20 and E1.25 + M20) than in the non-MDMA treated groups (p < 0.001, in all cases).

DA turnover showed a significant effect of the interaction Alcohol × Dose of MDMA [F (2,30) = 5.507; p < 0.01], with a decrease being observed in the groups treated only with EtOH (p < 0.05) or 10 mg/kg of MDMA (p < 0.02).

The concentrations of striatal [F (1,30) = 4.777; p < 0.03] and hippocampal [F (2,30) = 5.215; p < 0.03] serotonin showed an effect of the variable Alcohol with higher levels of this monoamine being detected in animals treated with EtOH (E1.25, E1.25 + M10 and E1.25 + M20).

## **Discussion**

This is the first study to address the long-lasting effects of intermittent administration of EtOH plus MDMA during adolescence on learning and memory in adult mice. Our results confirm that the adolescent brain is highly sensitive to the administration of EtOH and/or MDMA and that the effects of these drugs are manifested in adulthood. Exposure to EtOH or MDMA during adolescence, either separately or together, impaired learning in the Hebb-Williams maze. All the treatment groups required more time to reach the goal than controls. In addition, animals treated with EtOH presented longer latencies in the last four trials and did not show a reduction in the time needed to reach the goal in consecutive trials, unlike the groups treated with saline or MDMA alone. The groups treated with EtOH also required more trials to reach the acquisition criterion score. These results highlight that ethanol impairs the development of learning regardless of whether or not MDMA is also administered. However, a progressive reduction in the number of errors committed in successive trials was observed in all the groups, in both easy and difficult mazes. On the other hand, monoamine levels were significantly affected by treatment during adolescence, with a significant decrease of striatal DA being observed in the groups treated with 20 mg/kg of MDMA, alone or plus EtOH and in the groups treated with 10 mg/kg of MDMA plus EtOH, thus confirming that EtOH increases the neurotoxic effect of MDMA in mice.

The Hebb-Williams maze is a complex spatial learning test that is frequently used to detect changes in cognitive functions [43], such as those brought about by environmental enrichment [44]. One of the advantages of this test is that the mazes vary in difficulty, so that animals with learning disabilities perform more poorly as the tasks become more difficult. The five mazes employed in the present work were chosen on the basis of their varying levels of difficulty. Mazes 1, 3 and 4 are generally considered to be easy, while mazes 5 and 8 are considered to be difficult [45], although maze 5 has also been classified as intermediate [46]. The time needed for the animals to reach the goal or the acquisition criterion score and the number of errors was significantly higher in the difficult mazes than in the easy mazes, thus confirming their varying levels of difficulty.

Previous reports have failed to demonstrate a long lasting effect of MDMA administration on learning and memory. Thompson et al. [47] reported that prenatal exposure to MDMA did not affect performance in the radial arm maze or the Morris water maze. In line with those findings, and using the same doses and pattern of MDMA administration, we have previously failed to observe an effect on memory in the passive avoidance test in adult mice treated with MDMA during adolescence [20]. However, in the present study, the execution of the mazes was impaired by both of the MDMA doses employed. The lowest dose (10 mg/kg) produced a slight effect, as the total latency increased only in the easy mazes, and not in the difficult mazes. However, animals treated with 20 mg/kg of MDMA presented longer total latencies in both type of mazes. Thus, complex learning is affected by this pattern of MDMA administration. In a recent study [48] showed that rats exposed during adolescence to a combination of alcohol and MDMA exhibited significant memory deficits in the radial arm maze, although the authors did not observe a specific effect of alcohol or MDMA when either substance was administered alone, probably due to the used of low doses administered only two times.

We have previously described the deleterious effect of intermittent intensive EtOH ingestion during adolescence on learning and memory in rats [30]. The present findings confirm those results and, additionally, we have observed that the effect of EtOH is independent of that of MDMA. All the groups treated with EtOH presented a longer total latency to achieve the goal and needed more trials to reach the acquisition criterion score than those that did not receive EtOH, while the former animals did not present a reduction in latency in successive trials, unlike the latter. It should be pointed out that the dose of EtOH employed in the present study was smaller (1.25 g/kg) than that used by Pascual et al. (3 g/kg) and was chosen on the basis of other studies carried out in our laboratory [20] in which it did not induce strong behavioral effects when administered alone, thus allowing the interaction with MDMA to become apparent. Other authors have reported that low doses of EtOH (0.5 g/kg) do not impair learning, whereas high doses do, regardless of the age of the animal [49]. Moreover, chronic intermittent EtOH exposure during adolescence does not impair spatial learning when rats are trained on days on which they are not treated with EtOH [50,51]. In a binge paradigm, adolescent rats develop a higher tolerance to EtOH-induced loss of righting reflex than adults, and this tolerance is maintained in adulthood [50,52]. For some authors, these data suggest that the acquisition of spatial cognition in adolescent rats is resistant to chronic intermittent EtOH administration and that the adolescent hippocampus is not as fragile as previously indicated [53]. In contrast, our data confirm that administration of a low dose of EtOH in a binge pattern during adolescence induces long lasting effects on the learning of a complex task in adult animals.

Analysis of the different treatment groups revealed that all the animals committed fewer errors as the trial progressed, which is proof that they learned the task. However, latency data showed that all the groups, and especially those treated with EtOH, required more time than controls to reach the goal. Latencies can be affected by some variables such as motor activity or anxiety. Thus, to correctly interpret these data, we must take into account that the animals treated with 20 mg/kg of MDMA during adolescence, alone or plus EtOH, exhibited higher levels of locomotor activity in adulthood [20]. This effect led these animals to engage in more exploration of the maze and, thus, to commit more mistakes (especially in the first trial), although the number of errors decreased as the trials progressed. On the other hand, a high percentage of animals in the E.15 + M10 group (an average of 50%) exhibited immobility and did not reach the goal in the maximum time permitted (5 minutes) in either easy or difficult mazes. As they were less active, these animals committed fewer errors but presented the longest latencies to reach the goal. Although all the mice treated with EtOH plus MDMA displayed higher levels of anxiety, only those treated with 20 mg/kg also presented higher motor activity. The longer latencies observed in the E1.25 + M10 group could have been due, at least partially, to their elevated level of anxiety [20], which would have made them engage in less exploration behavior. On the other hand, the mice treated with EtOH plus the highest MDMA dose, though displaying similar levels of anxiety, were more active and explored the maze more (and committed more mistakes), which could have counteracted the anxiogenic effect and enabled them to reach the goal more quickly.

The results observed in this study could be affected by the different neurotoxic profiles of the two MDMA doses employed. In accordance with a previous report by our group [20], this pattern of administration did not induce neurotoxic damage when 10 mg/kg of MDMA were administered, but a significant decrease in striatal DA concentration was induced by 20 mg/kg, an effect that was augmented by EtOH. Striatal DA concentration was lower in the two groups treated with 20 mg/kg of MDMA and in that treated with 10 mg/kg of MDMA plus EtOH. DOPAC levels dropped in all the treatment groups, although the decrease was significant only in those treated with the highest dose of MDMA, which also displayed lower DA levels. In this way, the decrease in DA turnover occurred only in the groups treated with EtOH or 10 mg/kg of MDMA alone, which presented DA concentrations similar to those of the control group. Impairment of the learning processes and alteration of DA neurotransmission (a decrease in the metabolite concentration or its turnover) were observed in all the treatment groups, In addition, an increase in striatal and hippocampal serotonin was observed in the EtOH-treated groups (those which presented more affectation of learning), thus altering the equilibrium between these two neurotransmitters. Learning and memory depend, at least in part, on short- or long-lasting synaptic modifications that occur mainly at dendritic spines. The modulatory influence of 5-HT and DA at the synaptic level may affect the codification of mnemonic information in such spines. Several experimental models of neurotransmitter activity have identified a close association between an imbalance of 5-HT-DA and cytoarchitectonic changes underlying learning and memory impairment [54]. Evidence indicates that pharmacological disruption of serotonin neurotransmission promotes the processing of mnemonic information by cerebral regions subjected to strong DA modulation. On the other hand, increased serotonin neurotransmission appears to have a detrimental effect on the cognitive functions of these structures [55].

There are few studies to have evaluated the interaction between EtOH and a neurotoxic dose of MDMA. Exposure to a neurotoxic dose of MDMA has been shown to decrease the sedative-hypnotic effect of acute EtOH. In MDMA-lesioned mice, EtOH did not modify striatal GABA accumulation, suggesting that the regulation of GABAergic neurons is less sensitive to the effects of EtOH when a brain has been damaged by MDMA [56]. It should be taken into consideration that GABAA receptor-mediated inhibitory tonic currents in the dentate gyrus of the hippocampus are more enhanced in adolescent rats than in adults after EtOH administration [57], which highlights the importance of the changes in GABA function reported by Izco and coworkers (2010). In line with this, a previous report by the same authors showed that mice pre-exposed to a neurotoxic dose of MDMA exhibited a higher consumption of and preference for EtOH than saline-treated animals. These mice also exhibited a lower level of release of basal dopamine in the nucleus accumbens when compared with saline-injected animals. Intraperitoneal administration of EtOH produced an increase of extracellular dopamine release in the nucleus accumbens of saline-treated mice, but this effect was almost non-existent in MDMA-treated mice [14].

The interaction between EtOH and MDMA can be explained in different ways. The presence of EtOH increases the availability of MDMA in the plasma and brain of mice [15] and in the plasma of humans [58]. A recent study in rats showed that EtOH increases delivery of MDMA to the brain, especially in the striatum and cortex, an effect that could increase the risk of drug neurotoxicity [16]. Additionally, there seems to be an additive synergism between the effects of MDMA and EtOH on the release of monoamines, and particularly that of dopamine and 5-HT. In this context, a local synergistic interaction of EtOH and MDMA with the spontaneous outflow and electrically-evoked release of striatal DA and 5-HT has been reported [59]. In this way, when administered with a low dose of MDMA, the EtOH present in several brain areas can increase, thus enhancing the behavioral effects observed.

The use in animal studies of much higher doses and different routes of administration to those of recreational human use raises the question of whether animal data reflect ‘heavy’ use of MDMA [60]. A recreational user can be defined as ‘a person who ingests a standard dose (80–150 mg) of MDMA’ occasionally [61,62]. When extrapolated to humans, the doses and pattern of MDMA administration employed in this study represent a higher intake of ecstasy, but the marked metabolic differences between rodents and humans should also be taken into consideration [63]. Mice have a more rapid and efficient metabolism than humans and are not thought to possess an auto-inhibition of the metabolism of MDMA. Bearing in mind these differences, we chose a consecutive pattern of MDMA administration, since we calculated that 4 doses would induce lower levels of the drug in mice than in humans. In addition, we administered a moderate dose of EtOH. Therefore, our model mimics the pattern of use of adolescents who take a moderate or high (in case of 20 mg/kg) number of MDMA pills with 2 or 3 alcoholic drinks. The use of a mouse model to study the effects of MDMA has certain aspects that must be taken into consideration. This model is a useful tool to isolate the consequences of dopaminergic neurotoxicity, which is of great interest in light of the high number of MDMA consumers who also take methamphetamine. In general, studies of MDMA performed in animal models, especially those which focus on its neurotoxic effects, provoke serious concerns, regardless of the species employed. Most rodent studies employ an acute regimen of high doses of MDMA in order to induce neurotoxicity, whereas humans tend to extend their use over weeks. In addition, the dose of MDMA employed may exceed the normal dosage of human abuse. It is clearly difficult to directly compare the dosages that cause neurotoxicity in rodents and humans respectively; in addition to metabolic differences, poly-drug use and environmental stimuli can modulate the pharmacological effects of the drug in humans but not in rodents, which are maintained in a controlled laboratory environment. All of these factors make it impossible to directly extrapolate any finding from rodent studies to humans.

## **Conclusions**

The risks associated with multi-drug exposure during adolescence are still to be clarified. However, it is clear that the developing brain is highly vulnerable to the damaging effects of EtOH and that these effects are usually irreversible (for a review, see Guerri, 2002). Our study has confirmed this vulnerability and reveals that mice treated during adolescence with a binge pattern of EtOH, MDMA or both drugs together exhibit impaired learning and memory in both easy and difficult Hebb-Williams mazes in adulthood. Although this effect was independent of the MDMA-induced decrease in striatal DA concentration, all the affected groups displayed an imbalance in the DA/serotonin interaction.

## **Competing interests**

The authors declare that they have no competing interests.

## **Authors' contributions**

All the authors have made a substantial contribution to the conception and design of the study (JM, MAA and MR-A), the acquisition, analysis and interpretation of the data (AV-I and MR-A) and the drafting and revision of the article (AV-I, JM, MAA, and MR-A). All the authors have approved the present version for submission.
